# Calcimimetic acts on enteric neuronal CaSR to reverse cholera toxin-induced intestinal electrolyte secretion

**DOI:** 10.1038/s41598-018-26171-4

**Published:** 2018-05-18

**Authors:** Lieqi Tang, Lingli Jiang, Megan E. McIntyre, Ekaterina Petrova, Sam X. Cheng

**Affiliations:** 1 Department of Pediatrics, University of Florida, Gainesville, FL 32610 USA; 2 Department of Pediatrics, Division of Gastroenterology, Hepatology and Nutrition, University of Florida, Gainesville, FL 32610 USA

## Abstract

Treatment of acute secretory diarrheal illnesses remains a global challenge. Enterotoxins produce secretion through direct epithelial action and indirectly by activating enteric nervous system (ENS). Using a microperfused colonic crypt technique, we have previously shown that R568, a calcimimetic that activates the calcium-sensing receptor (CaSR), can act on intestinal epithelium and reverse cholera toxin-induced fluid secretion. In the present study, using the Ussing chamber technique in conjunction with a tissue-specific knockout approach, we show that the effects of cholera toxin and CaSR agonists on electrolyte secretion by the intestine can also be attributed to opposing actions of the toxin and CaSR on the activity of the ENS. Our results suggest that targeting intestinal CaSR might represent a previously undescribed new approach for treating secretory diarrheal diseases and other conditions with ENS over-activation.

## Introduction

Acute secretory diarrheal illnesses, such as cholera, affect millions of children and adults each year and morbidity and mortality from the large fluid and electrolyte losses remain high^1,2^. The extracellular calcium-sensing receptor (CaSR)^3^ is an ancient G protein-coupled cell surface receptor that is expressed in diverse tissues in mammalian^4^ and marine^5^ species and is a key regulator of tissue responses required for calcium homeostasis^3^, salt and water balance^6,7^, and osmotic regulation^5^. The primary physiological ligand for the CaSR is extracellular ionized calcium (Ca^2+^_o_), providing a mechanism for Ca^2+^_o_ to function as a first messenger. The CaSR also functions as a more general sensor of the extracellular milieu due to allosteric modification of Ca^2+^_o_ affinity by polyamines, _L_-amino acids, pH and ionic strength^8^.

Enteric mucosal cells along the entire small and large intestines express the CaSR. These include enterocytes^9–12^, neurons in the enteric nervous system (ENS)^9,13^ and enterochromaffin (EC) cells^14^ and other enteroendocrine (EE) cells (refer to a recent review by Tang *et al*.^15^ for refs). Studies that characterize the physiologic function of the CaSR in the gastrointestinal (GI) tract have just begun^15,16^. CaSR has been identified on both the apical and basolateral membranes of human^11,17^ and rat colonocytes^9,11^. Receptors localized to both membrane domains of this polarized epithelium are functionally active and can be activated by Ca^2+^_o_, calcimimetics such as R568, and other polycations such as spermine with similar potency and EC_50_ values^10,11^. In isolated microperfused colonic crypts, CaSR activation from either the mucosal or serosal side inhibited net fluid secretion^10,11,18^ and cyclic nucleotide accumulation^18^ induced by synthetic/natural secretagogues such as forskolin and guanylin, which generate cyclic AMP and cyclic GMP, respectively. CaSR activation also blocks the effects of bacterial enterotoxins^18^ such as cholera toxin (CTX), a potent activator of membrane-bound adenylyl cyclase leading to elevated intracellular levels of the cyclic AMP. It also blocks the effects of heat stable *Escherichia coli* enterotoxin (STa)^18^, which enhances cytosolic cyclic GMP accumulation through the guanylyl cyclase C-type guanylin receptor. CaSR has also been localized to the neurons in the ENS^9,13^, the brain of the gut^19^, and thus could regulate neural secretory responses. However, studies that characterize the role of CaSR in the enteric neurons have been limited.

In rodents and humans, the ENS, mainly the submucosal plexus, releases neurotransmitters [e.g., vasoactive intestinal peptide (VIP) and acetylcholine (ACh)] that regulate fluid secretion (see reviews^20–22^). There is compelling evidence from *in vivo* experiments that the ENS modulates intestinal fluid secretion induced by bacterial enterotoxins (e.g., CTX, STa)^21–23^, as well as by viral enterotoxins [e.g., rotavirus nonstructural protein 4 (NSP4)]^24,25^. For example, cholera toxin-induced fluid secretion is blocked by tetrodotoxin (TTX) or lidocaine, inhibitors of neural activity^26^, or hexamethonium, an inhibitor of cholinergic nicotinic receptor antagonist^27^. Based upon this, a dual pathway model for fluid secretion and diarrhea formation has been proposed: (1) a non-neuronal fluid secretory response due to binding of enterotoxins directly to enterocytes, leading to generation of cyclic nucleotides, which is TTX/lidocaine-insensitive; (2) a neuronal secretory response that is mediated by stimulation of the neurons in the ENS, which is TTX/lidocaine-sensitive. The calcimimetic R568 is a specific CaSR agonist. Using isolated rat colon segments containing intact ENS mounted in Ussing chambers, we have previously shown that R568, when applied serosally, inhibited basal and forskolin-induced secretory currents in the absence, but not presence, of TTX^13^. This suggests that this anti-secretory agent acts on the intestine through neurons within the ENS. Although the finding from this latter study on calcimimetic is consistent with the dual-pathway regulation model, it is also possible that R568’s anti-secretory effect results from its binding to the receptor in the basolateral membrane of mucosal epithelial cells. Furthermore, R568 might inhibit TTX/lidocaine-dependent secretory current indirectly, by acting on the CaSR on mucosal epithelial cells that then interact with TTX/lidocaine-sensitive enteric neurons. Finally, there is a small likelihood that R568’s anti-secretory effect is due to a non-specific off-target effect of this agent^28^.

To test the hypothesis that R568 acts on neuronal CaSR of the ENS to inhibit electrolyte secretion by the intestine, the present study employed a tissue-specific knockout mouse approach in conjunction with Ussing chamber methods for further studying the effect of this calcimimetic on basal and secretagogue-evoked intestinal secretion. In addition to forskolin, we also employed cholera toxin. We selected cholera toxin because it is a secretagogue known to activate enteric neuronal reflexes^21^. The two tissue-specific CaSR mutant mouse lines used were: (1) Mucosal epithelial cell CaSR-specific conditional knockout mice (^villin^*Cre/Casr*^flox/flox^ mice)^29^, and (2) Neuronal cell CaSR-specific conditional knockout mice (^nestin^*Cre/Casr*^flox/flox^ mice)^30^. Our results show that CaSR activity on enteric neurons is required for R568-mediated inhibition of cholera toxin-induced electrolyte secretion by the intestine. This is the first demonstration that the CaSR expressed by enteric neurons is functional. Thus, apart from their direct mucosal epithelial effects shown in our previous studies using ENS-absent colonic epithelial crypts^10,11,18^, our present data show that the pro- and anti-secretory effects of cholera toxin and R568 on electrolyte secretion by the intestine could also be mediated, indirectly, through opposing actions of the two agents on the activity of the ENS.

## Results

### Calcimimetic inhibits basal I_sc_ in mouse colon via CaSR on enteric neurons

**Figure 1:**
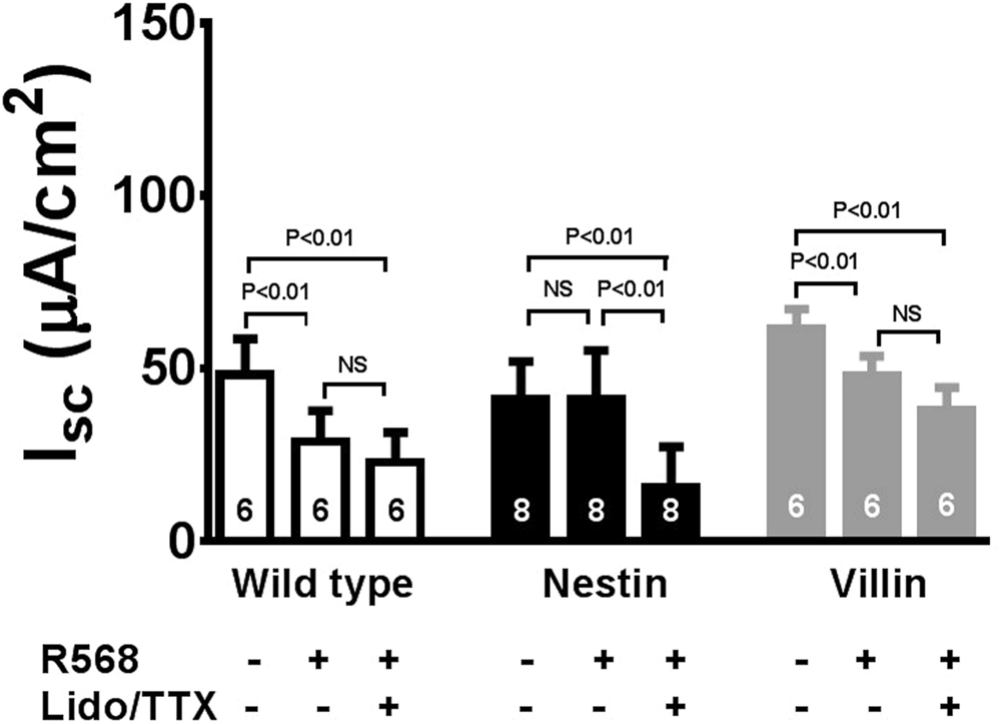
R568 anti-secretory effects under basal conditions. Summarized are steady-state I_sc_ changes in response to the sequential serosal additions of R568 (10 µM) and lidocaine (Lido, 1.6 mM)/TTX (2 µM). The R568 anti-secretory effect is present in colons of wild-type mice and mice expressing neuronal CaSR (^villin^*Cre/Casr*^flox/flox^ or villin mice) but is absent in mice lacking neuronal CaSR (^nestin^*Cre/Casr*^flox/flox^ or nestin mice). NS, no significance.

Under basal conditions, mucosal distension during normal digestion or mechanical stimulation during experimental preparations can activate neuronal reflex pathways in the ENS, evoking Cl^−^ secretion^20,31^. This reflex-evoked Cl^−^ secretion can be measured in Ussing chamber experiments as increases in TTX/lidocaine-sensitive short-circuit current (I_sc_)^13^. To test the hypothesis that CaSR is expressed on enteric neurons to restrict neurally mediated secretory responses, we generated a neuronal tissue-specific CaSR conditional knockout mouse line (i.e., ^nestin^*Cre/Casr*^flox/flox^ mice or nestin mice) using Cre recombinase. For comparison, we also generated an epithelial tissue-specific CaSR conditional knockout (i.e., ^villin^*Cre/Casr*^flox/flox^ mice or villin mice). Colons from these knockouts were isolated, mounted onto Ussing chambers, and Cl^−^ secretory I_sc_ responses to R568 were measured and compared with their wild-type littermates. The results are shown in Fig. 1. Consistent with the presence of neurogenic Cl^−^ secretion, a large TTX/lidocaine-sensitive I_sc_ was detected in these mouse colons. In wild-type mice, R568 (10 µM, added to the serosal bath) inhibited basal secretory I_sc_ and abolished the inhibitory effect of TTX/lidocaine (2 µM/1.6 mM, added to the serosal bath) on I_sc_. In neuronal CaSR knockout mice or nestin mice, however, R568 did not significantly inhibit I_sc_, although it caused significant inhibition on I_sc_ in epithelial CaSR knockout mice or villin mice. In all three groups, inhibitory effects of TTX/lidocaine on basal I_sc_ were unaffected [mean % inhibition of I_sc_ by TTX/lidocaine in wild-type, nestin and villin mice: 55 ± 12% (6), 63 ± 7% (8) and 43 ± 8% (6), P > 0.05]. These results suggest that the calcimimetic acts on CaSR of enteric neurons, not mucosal cells, to inhibit TTX/lidocaine-sensitive electrolyte secretion by the intestine.

### Calcimimetic inhibits cholera toxin-evoked I_sc_ in mouse colon via CaSR on enteric neurons

**Figure 2:**
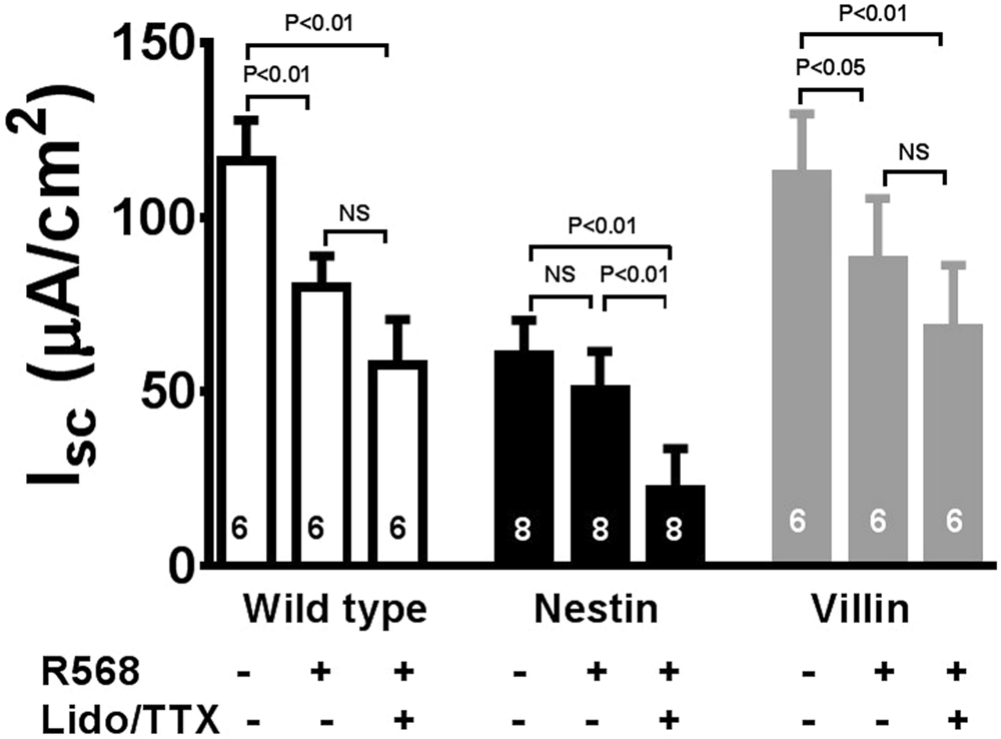
R568 anti-secretory effects under cholera toxin-stimulated conditions. Mouse intestines were pretreated with cholera toxin, *in vivo*, in live animals to induce hypersecretion (see Methods), followed by examining I_sc_ changes of pre-treated intestines, *ex vivo*, in Ussing chambers. Summarized are steady-state I_sc_ changes of these pretreated intestines in response to the sequential serosal additions of R568 (10 µM) and lidocaine (Lido, 1.6 mM)/TTX (2 µM). The R568 anti-secretory effect is present in colons of wild-type mice and mice expressing neuronal CaSR (^villin^*Cre/Casr*^flox/flox^ or villin mice) but is absent in mice lacking neuronal CaSR (^nestin^*Cre/Casr*^flox/flox^ or nestin mice). NS, no significance.

To strengthen the hypothesis that R568 acts on CaSR of enteric neurons to limit secretion, we also performed experiments using cholera toxin to enhance the neurally mediated secretory state. In a pilot study, we found that mouse colons did not survive the long (90 min) incubation required for cholera toxin to have an effect in Ussing chambers. Accordingly, we adopted a two-step protocol previously described by Gabriel *et al*.^32^, i.e., pretreating mouse intestines with cholera toxin, *in vivo*, in live animals to induce hypersecretion (see Methods), followed by examining I_sc_ responses of pre-treated colons, *ex vivo*, in Ussing chambers (Fig. 2). Compared with non-cholera toxin controls, cholera toxin pretreatment significantly increased I_sc_ in colons of wild-type mice and both knockouts (see Table 1). Subsequent treatment with R568 abrogated cholera toxin-induced increases in I_sc_ in wild-type and villin mice, but not in nestin mice. In all three groups, inhibitory effects of TTX/lidocaine on cholera toxin-augmented I_sc_ were unaffected [mean % inhibition of I_sc_ by TTX/lidocaine in wild-type, nestin and villin mice: 51 ± 8% (6), 62 ± 11% (8) and 40 ± 12% (6), P > 0.05]. These results confirm the finding in Fig. 1, suggesting that this calcimimetic anti-secretory agent acts on CaSR of enteric neurons, not mucosal cells, to reduce cholera toxin-induced electrolyte secretion by the intestine.

**Table 1:** I_sc_ responses to cholera toxin in wild-type and CaSR mutant mouse colons.

	I_sc_ (µA/cm^2^)		
	Wild-type	^nestin^ *Cre/Casr* ^flox/flox^	^villin^ *Cre/Casr* ^flox/flox^
Control	48 ± 10 (6)	41 ± 10 (8)^nsNS^	65 ± 6 (6)^ns^
Cholera toxin	116 ± 12 (6)**	60 ± 10 (8)*^,##,¶¶^	113 ± 17 (6)**^,ns^

Data shown are means ± SEM (n).

**P < 0.01, *P < 0.05 *vs*. Control.

^##^P < 0.01, ns > 0.05 *vs*. Wild-type.

^¶¶^P < 0.01, NS > 0.05 *vs*. ^villin^*Cre/Casr*^flox/flox^.

### Calcimimetic inhibits forskolin-evoked I_sc_ in mouse colon via CaSR on enteric neurons

**Figure 3:**
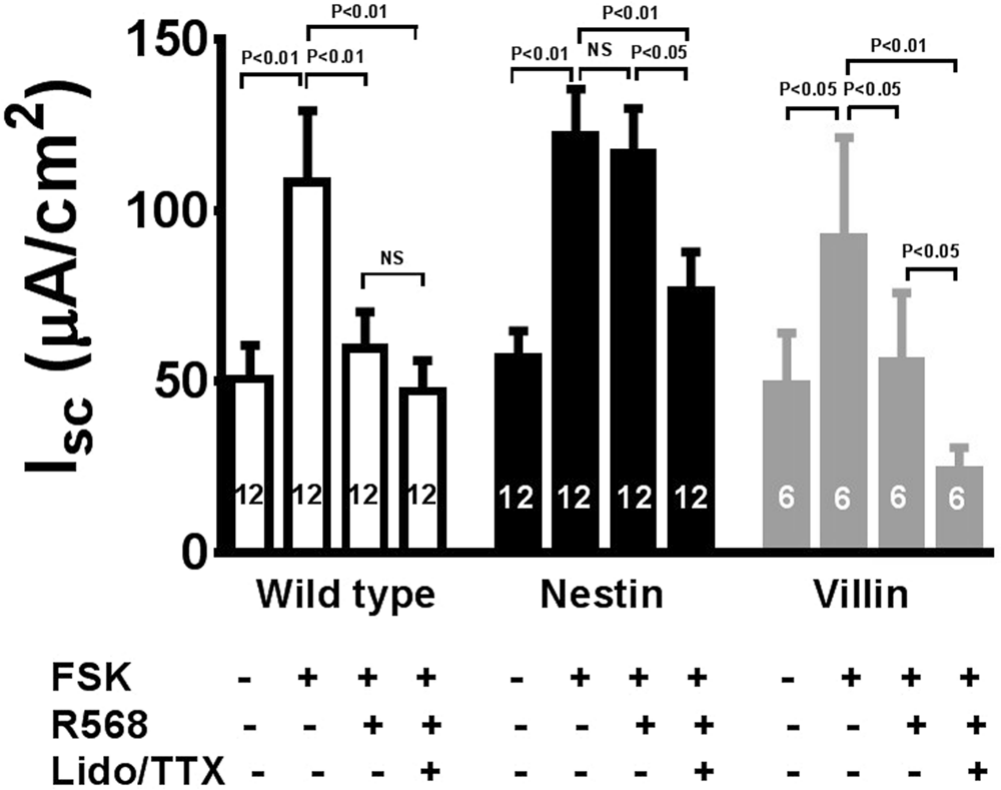
R568 anti-secretory effects under forskolin-stimulated conditions. Mouse intestines were pretreated with forskolin (FSK, 500 nM, added to the serosal bath), *ex vivo*, in Ussing chambers to elevate cyclic AMP and excite neurons to induce hypersecretion. Following this pre-treatment, R568 (10 µM) and lidocaine (Lido, 1.6 mM)/TTX (2 µM) were subsequently added to the serosal solution of Ussing chambers with the sequence of their additions indicated. Summarized are steady-state I_sc_ changes of these pretreated intestines and effects of R568 and lidocaine/TTX. R568 abolishes the forskolin’s excitatory effect in colons of wild-type mice and mice expressing neuronal CaSR (^villin^*Cre/Casr*^flox/flox^ or villin mice) but not in mice lacking neuronal CaSR (^nestin^*Cre/Casr*^flox/flox^ or nestin mice). NS, no significance.

In colonic crypts^18^ as well as in kidney^33^ and parathyroid cells^34^, CaSR exerts its regulatory actions via reductions of the cyclic AMP. Thus, to provide insights into the messenger systems involved in the actions of the CaSR in enteric neurons, we also studied effects of R568 on forskolin-induced I_sc_ (Fig. 3). We chose forskolin also because in enteric neurons this agent has long been shown to simulate the neuroexcitatory effects of 5-hydroxytryptamine (5-HT), substance P, VIP and many other neurotransmitters released in neuronal reflexes^35^. Indeed, forskolin (500 nM, added to the serosal bath) significantly increased I_sc_ in colons of wild-type mice and both knockouts. Subsequent addition of R568 significantly diminished forskolin-induced increases in I_sc_ in wild-type mice and villin mice, but not in nestin mice. In all three groups, inhibitory effects of TTX/lidocaine on forskolin-evoked I_sc_ were unaffected [mean % inhibition of I_sc_ by TTX/lidocaine in wild-type, nestin and villin mice: 52 ± 6% (12), 36 ± 11% (12) and 67 ± 8% (6), P > 0.05]. These results suggest that activation of adenylate cyclase by forskolin and subsequent elevation of intra-neuronal cyclic AMP mimic the effects of cholera toxin-induced neuronal cell excitation on Cl^−^ secretion, thus supporting the possibility that reductions of cyclic AMP might be involved in the actions of this neural CaSR.

### Neural CaSR is required for enteric neurons to communicate with the mucosal epithelium

Enteric neurons communicate with the mucosal epithelium in not one- but two-way directions. In addition for the mucosa (e.g., EC cells) secreting neuroactive agents (e.g., 5-HT) to control the activity of enteric neurons, enteric neurons also release neurotransmitters (e.g., VIP, somatostatin) to influence the function of the mucosal cells (e.g., EC cells)^36,37^. Thus, after characterizing its role in regulating the functionality of enteric neurons, we asked if CaSR on enteric neurons also regulates the functions of the mucosal epithelium. For this, we compared I_sc_ changes in response to cholera toxin (which was administered to the mucosal side) and forskolin (which was administered to the serosal side). The rationale is that cholera toxin requires mucosa to activate a secretory neural reflex whereas forskolin does not. Accordingly, if CaSR on enteric neurons regulated the functionality of the mucosa, then cholera toxin should increase I_sc_ differently in nestin mice compared with wild-type and villin mice, whereas forskolin should have a similar effect on I_sc_ in all three groups. Indeed, although both pro-secretory agents increased I_sc_ in all three animal groups, cholera toxin increased I_sc_ to a significantly lesser extent in nestin mice (Fig. 2 and Table 1), whereas forskolin had a similar effect on I_sc_ in all three groups (Fig. 3 and Table 2). Thus, in nestin mice, the mean % increase in I_sc_ induced by cholera toxin was only 20 ± 12% (8), which is significantly smaller than 141 ± 59% (6) in wild-type (P < 0.05) and 83 ± 28% (6) in villin mice (P < 0.05). In contrast, the mean % increases in I_sc_ induced by forskolin were 126 ± 25% (12), 120 ± 23% (12), and 86 ± 29% (6), respectively (P > 0.05). These results suggest that, in addition to regulating neuronal function, the CaSR expressed by neuronal cells also modulates the function of the mucosa, highlighting the importance of this receptor in GI physiology and pathophysiology.

**Table 2:** I_sc_ responses to forskolin in wild-type and CaSR mutant mouse colons.

	I_sc_ (µA/cm^2^)		
	Wild-type	^nestin^ *Cre/Casr* ^flox/flox^	^villin^ *Cre/Casr* ^flox/flox^
Control	50 ± 10 (12)	57 ± 8 (12)^ns^	49 ± 15 (6)^ns^
Forskolin	108 ± 21 (12)**	122 ± 14 (12)**^,ns,NS^	92 ± 29 (6)*

Data shown are means ± SEM (n).

**P < 0.01, *P < 0.05 *vs*. Control.

ns P > 0.05 *vs*. Wild-type.

NS P > 0.05 *vs*. ^villin^*Cre/Casr*^flox/flox^.

## Discussion

Our data confirm that CaSR in enteric neurons is functional and can modulate neurogenic secretory responses. Using a rat colon model, we have previously shown that the serosally applied calcimimetic R568 inhibits forskolin-induced secretory I_sc_ in the absence, but not presence, of the neurotoxin TTX^13^, suggesting that this anti-secretory agent might act on the intestine via enteric neurons within the ENS. In the present study, we extend these observations by showing that in mouse colons this same small molecule compound also inhibits basal and secretagogue (forskolin and cholera toxin)-induced I_sc_ in a neuronal CaSR-dependent manner, i.e., R568 inhibited basal or evoked secretory I_sc_ in colons of wild type mice and mutant mice that contained CaSR in neurons (i.e., ^villin^*Cre/Casr*^flox/flox^ mice), but not in colons of ^nestin^*Cre/Casr*^flox/flox^ mice in which CaSR in neuronal cells was eliminated, providing further support for potential roles that this neuronal CaSR may play in the control of ENS activity and GI function.

The primary function of the GI tract is to digest food and absorb nutrients. To aid in digestion, the GI tract secretes fluid. It is estimated that following the intake of a meal, intestinal secretion can be increased eightfold^38^. There is compelling evidence that the ENS has a major role in enhancing this secretory state^20^. This enhanced secretion helps lubricate the surface of the lumen of the intestine, ensuring the appropriate mixing and flow of digest along its length. Once digestion is completed and nutrients are extracted, these secretions along with released nutrients must be reabsorbed while also ensuring that further post-digestive secretions do not occur. These processes are highly regulated and coordinated; failure to do so may result in diseases such as mal-digestion, mal-absorption, constipation, or diarrhea. How these functions are regulated and coordinated remains a focus of study. We have presented evidence from this and other studies that CaSR may play a major role in the control of these processes (see a recent review by Tang *et al*.^15^). CaSR is a highly conserved G protein-coupled cell surface receptor (GPCR) that is coupled to multiple G proteins including G_q/11_, G_i/o_, and G_12/13_, and its downstream factors, and, when activated, turns on various signaling pathways regulating cellular behaviors. These include an increase of intracellular Ca^2+^, alteration of the intracellular cyclic AMP, phosphorylation of ERK1/2, and activation of small GTPase RhoA^39^. However, CaSR is an unusual GPCR in that it uses extracellular nutrients (e.g., calcium, polyamines, certain amino acids and peptides) as its ligands. Accordingly, unlike most other GPCRs, CaSR must function in the chronic presence of agonists. This is possible because CaSR adopts unusual mechanisms to regulate receptor expression, trafficking, and degradation^40^. For example, instead of inducing internalization and causing desensitization in other GPCRs, continuous elevation of agonist increases the number of CaSR in the plasma membrane via the so-called agonist-driven insertional signaling and sensitizes receptor function^40^. This is important because this unique property enables CaSR the ability to continuously monitor the ionic and nutritional compositions of the extracellular milieu in the gut and modulate the intestinal secretion/absorption in accordance with the status of digestion. Accordingly, local nutrient signals released from digestion, such as calcium, amino acids, and polyamines, can act on CaSR on neurons to function as a negative modulator of these physiological secretory actions and provides a mechanism for modulating net fluid movement during normal digestion. Based on this, we predict that over-activation of CaSR in this process may contribute to constipation whereas under-activation of CaSR may contribute to diarrhea. Indeed, people with high-calcium diets or taking excessive calcium are often constipated^41^, as are patients with hypercalcemia^42^. By contrast, those who are starved tend to hypersecrete in their small and large intestines^43–49^.

It is worth noting, however, that although this present study shows the primary effect of R568 is via neuronal CaSR, part of the inhibitory effect of this compound may be due to an action on epithelial CaSR. The latter is evidenced by the following: First, R568 produced a significantly less pronounced inhibitory effect on basal I_sc_ in ^villin^*Cre/Casr*^flox/flox^ knockouts than wild type mice [compare right *versus* left panels of Fig. 1; mean inhibition: 24 ± 5% (6) *versus* 40 ± 5% (6); P < 0.05], even though such a difference on cholera toxin [compare right *versus* left panels of Fig. 2; mean inhibition: 24 ± 7% (6) *versus* 29 ± 4% (6); P > 0.05] or forskolin stimulated I_sc_ [compare right *versus* left panels of Fig. 3; mean inhibition: 40 ± 5% (6) *versus* 40 ± 5% (12); P > 0.05] was not statistically significant. Second, R568 reduced forskolin-evoked I_sc_ and this clearly diminished the inhibitory effect of subsequent TTX/lidocaine addition in wild-type mice [Fig. 3 left panel; mean inhibition by TTX/lidocaine: 22 ± 5% (12)]. However, the ability of R568 to reduce the inhibitory effect of TTX/lidocaine was significantly less pronounced in ^villin^*Cre/Casr*^flox/flox^ knockouts (Fig. 3 right panel; mean inhibition by TTX/lidocaine: 46 ± 10% (6); P < 0.05). This suggests that epithelial CaSR may be involved, albeit to a lesser extent, in the inhibitory effect of R568.

Also noted was the finding that R568 failed to inhibit basal and secretagogue-induced I_sc_ in colons of ^nestin^*Cre/Casr*^flox/flox^ mice (Figs 1–3, middle panels). The reason is unknown. One possibility is the thickness of the tissue preparations used, which may prevent this hydrophobic calcimimetic drug from diffusing into the epithelium layer to access to the receptor^50,51^. An alternative explanation would be that the receptor function in the enterocyte might also be lost or its level may be down-regulated in this neuron-specific mutant mouse line. In support of this possibility, Crone *et al*. recently showed that the use of ^*nestin*^*Cre* mice also drove expression in the colonic epithelium^52^. Accordingly, the expression of *Casr* gene in the colonic epithelium may be reduced in ^nestin^*Cre/Casr*^flox/flox^ knockouts. Finally, epithelial stem cell production of the many different types of epithelial cells is under neural control so that any mechanism that alters neural activity can be expected to modify the epithelium and whether this affects EC or EE cell numbers or properties is not known. The CaSR knockouts used here are whole of life effects and hence it might be expected that there are significant differences in neural regulation of epithelial differentiation and hence insensitivity to calcimimetics. This may also explain their reduced sensitivity to cholera toxin (see below).

What is the most surprising was the finding on the effect of cholera toxin in ^*nestin*^*Cre/Casr*^*flox/flox*^ mice. In these animals, cholera toxin increased I_sc_ to a significantly lesser extent than it did in wild-type and ^*villin*^*Cre/Casr*^*flox/flox*^ (Fig. 2 and Table 1). This suggests that this neural CaSR may also influence the function of the mucosa, particularly the EC cells. Generating an effective secretory response to cholera toxin requires not only activation of secretomotor reflexes but also release of 5-HT from EC cells^53^. Thus, two possibilities may exist in ^*nestin*^*Cre/Casr*^*flox/flox*^ intestine: a defect occurred in the ENS or in the mucosa. The fact that only cholera toxin (which acts in the mucosa and ENS) but not forskolin (which acts in the ENS) caused the blunted I_sc_ response seems to suggest the latter, rather than the former. Indeed, via synaptic connections, the mucosa (e.g., EC cells) communicates with enteric neurons in a not mono- but bi-directional manner^54^. In addition for EC cells secreting neuroactive agents (e.g., 5-HT) to control the activity of enteric neurons, enteric neurons also release neurotransmitters to influence the function and fate of EC cells. Numerous receptors for neurotransmitters have been shown to be present on the basolateral aspect of EC cells^36,37^. When stimulated, these receptors modify the release of 5-HT. While some receptors (e.g., GABA and somatostatin) inhibit, others (e.g., VIP/PACAP) enhance, 5-HT release. Thus, neurogenic influences on EC cells may be altered in ^*nestin*^*Cre/Casr*^*flox/flox*^ intestines such that the balance is shifted to more inhibitory in these animals. In addition, EC cell proliferation and differentiation are under neural control. For example, enteric neurons may release PACAP/VIP to stimulate EC cell proliferation^55^. Thus, without influences from neuronal CaSR, EC cells may become hypoplastic and/or hypotrophic. Future studies need to characterize what exact changes occur to EC cells in ^*nestin*^*Cre/Casr*^*flox/flox*^ intestines and understand how CaSR elimination from enteric neurons leads to such changes.

The mechanisms by which CaSR inhibits the ENS activity are currently under investigation. In a recent study, Sun *et al*. examined the R568 effect on the myenteric neuron c-fos expression, a biochemical measure of neuronal excitability, and found that activation of CaSR by R568 significantly reduced neuronal cell c-fos expression in colons of wild-type mice and ^villin^*Cre*/*Casr*^flox/flox^ knockouts, but not ^nestin^*Cre*/*Casr*^flox/flox^ knockouts^30^. Thus, one possibility would be to operate by reducing neuronal excitability. Consistent with this, we show in the present study that activation of CaSR on enteric neurons inhibited the neurogenic secretion caused by the neuroexcitatory agent forskolin (Fig. 3). Forskolin is known to excite enteric neurons via activation of neuronal membrane adenylyl cyclase and subsequent elevation of the intra-neuronal cyclic AMP, which inhibits Ca^2+^ influx and closes Ca^2+^-dependent K^+^ channels, causing membrane depolarization^35^. Thus, CaSR might inhibit neuronal excitability by reversing this cellular process through receptor-mediated inhibition of adenylyl cyclase and/or reduction cyclic AMP, as described in enterocytes^10,11,18^ and many other cell types^39^. Recent studies on CaSR mechanisms on neurons of the CNS also seem to support this possibility^56^.

Alternatively, CaSR, particularly those expressed on nerve terminals of the ENS, may exert its inhibitory effect on neurons through inhibition of voltage-gated sodium channels (Na_v_) and thereby synaptic transmission and neurotransmitter release, as in the CNS^56^. Decreases in Ca^2+^_o_, a primary ligand of CaSR, have long been recognized to increase the likelihood of action potential initiation^57^. The CaSR location in nerve terminals of enteric neurons^13^, the TTX/lidocaine-sensitive nature of the CaSR-mediated suppression of neurogenic secretion^13^ (present study), and the abolition of the veratridine-evoked I_sc_ by R568 (Tang, unpublished observations) also seem to suggest this possibility. In the CNS, cyclic AMP regulates excitability/neurotransmission in two different pathways: action potential-dependent, which involves PKA-dependent phosphorylation and activation of Na_v_^58^, and action potential-independent, which involves PKA-independent Epac-dependent modifications of other mechanisms such as increasing Ca^2+^ influx^59,60^. Currently, it is unknown which pathway (s) is utilized by CaSR in the ENS.

Synaptic excitation is triggered by increases in intracellular Ca^2+^, which not only leads to the release of neurotransmitters but also increases synaptic H^+^ (co-released with neurotransmitters from vesicles) and Ca^2+^ (resulting from Ca^2+^/H^+^ exchange mediated via presynaptic plasma membrane Ca^2+^-ATPase)^61^. While the primary function of neurotransmitters is to excite postsynaptic effector cells, we do not know the roles and fates of these ions. The finding that enteric neuronal activity was inhibited by calcimimetics revealed in the present and the previous studies^30^ leads us to speculate that these synaptic Ca^2+^ and H^+^ ions may represent an internal ‘brake’ to limit synaptic excitation. Considering the known effects on CaSR of Ca^2+^_o_ (which activates receptor) and H^+^ (which inhibits receptor via allosteric modification of Ca^2+^_o_ affinity)^8^, we further hypothesize that CaSR is the receptor that detects and transduces changes in these synaptic ions into changes in cellular excitability. Furthermore, the resultant changes in cellular activity can be of either the presynaptic neuron or the postsynaptic neuron/epithelial cell or both.

The finding of the present study may have important pathophysiological significances. Bacterial enterotoxins, such as cholera toxin and STa, enhance intestinal fluid secretion and cause severe diarrhea. They do so through both direct enterocyte generation of cyclic nucleotides and indirect stimulation of the ENS to release neurotransmitter secretagogues such as VIP. Remarkably, our studies show that nearly all the secretory responses induced by cholera toxin are reversed upon activation of CaSR, acting either on the enterocyte^10,11,18^ or on the ENS (present study). Based on these findings, we predict that whether a patient develops diarrhea or not is determined not only by the toxin that provokes it but also by the activity of CaSR that prevents it. We also predict that, when this CaSR-mediated anti-secretory protection is decreased or lost, such as in children with a negative calcium balance or nutrient deprivation, this and other diarrheal diseases would be anticipated to be more common. Additionally, the diarrheal symptoms would be more severe, and the disease duration would be longer, as shown^62–64^. This CaSR protection theory may also explain why increasing calcium intake is effective in reducing the severity and duration of diarrhea resulting from enterotoxigenic *Escherichia coli*^65^ and other enterotoxin-producing infections^66^.

Effectively reducing the life-threatening intestinal fluid loss in enterotoxin-induced diarrhea remains a major challenge. The novel pathway for modulating intestinal Cl^−^ secretion through the colonic CaSR may lead to new pharmaco-nutritional therapies for prevention or treatment of certain clinical diarrheal diseases (i.e., cholera and other cyclic nucleotide-associated diarrheal diseases). Although the present study was conducted in the colon, the finding should apply to the small intestine, as CaSR is similarly expressed in the epithelium^9–12^ and neurons^9,13^ of the small and large intestines. Similarly, the primary virulence factor and diarrhea inducer of the *Vibrio cholerae* cholera toxin affects both the small^21–23^ and large intestine (present study), including the human colon^67^, even if the non-invading pathogen colonizes primarily the small intestine. Given that ENS-mediated secretory response is also critically implicated in many other forms of diarrhea, including viral [e.g., rotavirus^68^], neurogenic [e.g., irritable bowel disease^69^], and immunogenic/inflammatory diarrhea [e.g., inflammatory bowel disease^70^], the CaSR-mediated inhibition of the ENS-dependent secretory response observed in the present study might be of clinical importance in treating those forms of diarrhea as well.

**Figure 4:**
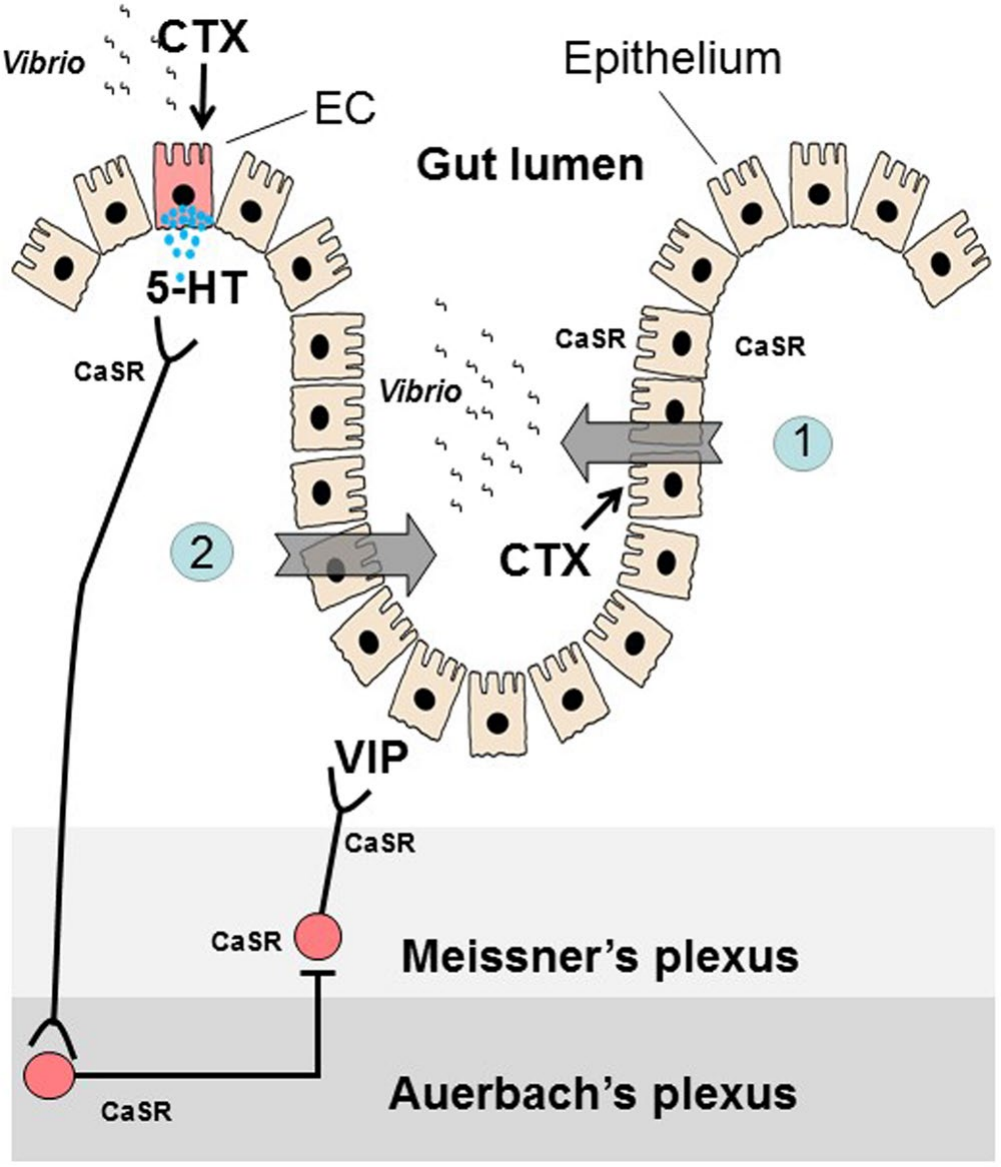
Proposed dual-pathway model for CaSR inhibition of cholera toxin-induced hypersecretion in the intestine. *Vibrio cholerae* generates cholera toxin (CTX) to evoke hypersecretion using two pathways and CaSR agonists block both pathways. (1) CTX induces a non-neuronal fluid secretory response due to binding of toxin directly to enterocytes, leading to the generation of cyclic AMP and activation of CFTR and NKCC1. This direct secretory response is blocked by activation of CaSR on enterocytes, either apically or basolaterally, through enhancing cyclic nucleotide destruction. (2) CTX induces a neurally mediated secretory response by stimulation of secretomotor reflex pathways in the ENS. This neurally mediated indirect secretory response is blocked by activation of CaSR on neurons (cell bodies, indicated in filled red circles, and nerve terminals, indicated in black lines) possibly by modulations of cyclic nucleotide metabolism and ion channel properties, leading to inhibition of neuronal cell excitability and/or synaptic excitation (see text for more discussions). Besides enterocytes and neurons, the cholera toxin-induced hypersecretion requires the release of 5-hydroxytryptamine (5-HT, indicated by blue dots) from enterochromaffin (EC, indicated in red) cells of the mucosa, which also express CaSR. It remains to be addressed whether cholera toxin-evoked release of 5-HT from EC cells is blocked by CaSR on EC cells.

In light of the present study and previously published data^10,11,13,18,29^, we propose a new paradigm for CaSR inhibition of cholera toxin-induced hypersecretion (Fig. 4). According to this paradigm, cholera toxin produces its effect on electrolyte secretion by the intestine using two pathways and CaSR agonists block both pathways. First, cholera toxin induces a non-neuronal fluid secretory response due to binding of toxin directly to enterocytes, leading to the generation of the cyclic AMP, and this direct diarrhea-causing pathway is inhibited by activation of CaSR on enterocytes. Second, cholera toxin induces a neuronal secretory response by stimulation of enteric neurons in the ENS, and this indirect diarrhea-causing pathway is inhibited by activation of CaSR on these neurons. Besides enterocytes and neurons, the cholera toxin-induced hypersecretion also requires the release of 5-HT and other mediators from EC cells, and EC cells express CaSR. Future studies need to address whether the cholera toxin-evoked release of 5-HT from EC cells is blocked by activation of EC cell CaSR. Nonetheless, the ability of CaSR agonists to reduce cholera toxin-induced fluid secretion demonstrated in this and other studies highly suggests that this class of nutrients and drugs may provide a unique therapy for cholera and other secretory diarrheal diseases.

## Materials and Methods

### Animals

Experiments were performed using non-fasting male/female C57BL/6 mice (wild-type and *Casr* mutants). Mice lacking CaSR expression in intestinal epithelial cells (^villin^*Cre/Casr*^flox/flox^ mice) and mice lacking CaSR expression in intestinal neurons (^nestin^*Cre/Casr*^flox/flox^ mice) and their wild-type littermates were bred and maintained in-house at the University of Florida Communicore Animal Facility. Mutant ^villin^*Cre/Casr*^flox/flox^ mice and ^nestin^*Cre/Casr*^flox/flox^ mice were generated as previously described^30,71^. Briefly, CaSR flox/flox mice were bred with transgenic mice expressing Cre Recombinase under the control of the villin 1 or nestin promoter and genotyped prior to all experiments after an approximate 10–12 generations. Mice were used at 5–10 weeks of age in accordance with the Animal Welfare Act and the Public Health Policy on Humane Care. Animals were fed and maintained on regular chow (Harlan) with free access to water before sacrifice. In some experiments, mice were induced for hypersecretion by cholera toxin (10 µg in 100 µl 7% NaHCO_3_ administered via intra-gastric gavage^72^) 6 hrs prior to sacrifice. Cholera toxin-induced hypersecretion/diarrhea was confirmed by the formation of loose stool and/or accumulation of clear fluid in the intestines as previously described^32,72–74^. Animals were sacrificed with standard CO_2_ inhalation and killed by cervical dislocation before colons were removed. The use of animals, as well as the protocols for cholera toxin treatment and colon tissue isolation, was approved by the Institutional Animal Care and Use Committee (IACUC# 201307567) at University of Florida.

### *Ussing chamber I*_sc_ measurements from colonic segments

Segments of colons were quickly isolated. Segments were cut open along the mesenteric border into a flat sheet and flushed with ice-cold basal HEPES-Ringer solution containing 1.25 mM Ca^2+^ and Mg^2+^. Intact full-thickness segments containing all the layers of middle colons (between proximal and distal colons) were used. Proximal and distal colons were distinguished grossly by the presence of oblique mucosal folds in the former and longitudinal folds in the latter. In a pilot study, we tested proximal and distal colons separately and found no significant difference in their *I*_sc_ responses to R568. The intestinal segments were then mounted between two halves of a modified Ussing chamber (Physiologic Instruments, San Diego, CA) and short-circuited continuously by a voltage clamp (VCC MC6, Physiologic Instruments, San Diego, CA) with correction for solution resistance. The exposure area was 0.3 cm^2^. The mucosal and serosal surfaces of the tissue were bathed in reservoirs with 3–5 ml HEPES-Ringer solution containing 110 mM NaCl, 5 mM KCl, 1.25 mM CaCl_2_, 1.25 mM MgCl_2_, 10 mM glucose, and 22 mM Hepes, pH 7.4, maintained at 37 °C and continuously bubbled with 100% O_2_. On average, an interval of 10 min elapsed between euthanizing the animal and mounting the tissue into the chamber. Tissues were allowed a minimum of 15-minute stabilization and basal recording period before test reagents or vehicles were added to the mucosal and/or serosal sides of the intestine. Trans-epithelial potentials generated by the tissues were continuously clamped to 0 mV, except for brief interruption to recording open-circuit potential (V_T_, mV). Tissue conductance (G_T_, mS/cm^2^) was calculated as the ratio of the measured short-circuit current (I_*sc*_, μA/cm^2^) to V_T_ from Ohm’s law. Data were acquired via DATAQ™ instruments and were stored on a PC and processed using the program Acquire & Analyze™. I_*sc*_ is defined as the current flow through the tissue when the tissue is short-circuited (i.e., when the voltage across the tissue is zero). Magnitudes (μA/cm^2^) of change in I_*sc*_ were determined before and after additions of test reagents or vehicle, with a positive sign representing net cation absorption and/or net anion secretion, and % changes in I_*sc*_ were calculated. In the present study, a two-step protocol was employed to induce hypersecretion by cholera toxin. To estimate the % changes in I_*sc*_ induced by cholera toxin, the basal I_*sc*_ from colons that were not pretreated with cholera toxin were pooled and used as controls.

In a pilot study, it was found that I_sc_ in mouse colons was less sensitive to TTX. Thus, in studies involving mouse colons, TTX was used in combination with lidocaine, another inhibitor of neural activity^26^. For measurements of TTX/lidocaine-sensitive neurally mediated I_*sc*_, 2 μM TTX and 1.6 mM lidocaine were applied to the serosal solutions and the differences in stable I_*sc*_ before and after TTX/lidocaine treatment were compared and calculated and were defined as TTX/lidocaine-sensitive I_*sc*_. According to a pilot experiment in which a dose-response effect on I_*sc*_ by TTX/lidocaine was sought, these doses of TTX and lidocaine were the lowest concentrations that maximally blocked the activity of the ENS without directly affecting the function of the epithelium^13,18,25^. Previous studies using Cl^−^ free Ringer solutions and pharmacologic inhibitors established that under present experimental conditions both the basal and secretagogue-stimulated I_*sc*_ primarily reflected Cl^−^ secretion^13^.

### Chemicals

Forskolin, veratridine, cholera toxin and lidocaine were obtained from Sigma, and stock solutions were prepared in dimethyl sulfoxide (DMSO) (forskolin, veratridine) or in water (cholera toxin). TTX was purchased from Enzo Life Science (Plymouth Meeting, PA), and 2 mM stock solutions were prepared in 10 mM acetic acid. R568 was purchased from Tocris Bioscience (Ellisville, MI), and 100 mM stock solutions were prepared in DMSO.

### Statistical Analysis

Values are given as means ± SEM of *n* experiments. Statistical comparisons between two means were performed by Student’s *t*-test, whereas comparisons among multiple means were by one-way ANOVA with Tukey’s post hoc tests. Both tests were performed either using Microsoft Excel 2016 for Windows or using GraphPad Prism version 6.07 for Windows (GraphPad Software, San Diego, CA). *P* < 0.05 was considered significant.

### Data availability

The datasets generated during and/or analyzed during the current study are available from the corresponding author on reasonable request.

## References

[1] Black R (2008). Global, regional, and national causes of child mortality in 2008: a systematic analysis. Lancet.

[2] Moore S, Lima AAM, Guerrant R (2011). Infection: Preventing 5 million child deaths from diarrhea in the next 5 years. Nat. Rev. Gastroenterol. Hepatol..

[3] Brown EM (1993). Cloning and characterization of an extracellular Ca(2+)-sensing receptor from bovine parathyroid. Nature.

[4] Brown EM, MacLeod RJ (2001). Extracellular calcium sensing and extracellular calcium signaling. Physiol. Rev..

[5] Nearing J (2002). Polyvalent cation receptor proteins (CaRs) are salinity sensors in fish. Proc. Natl. Acad. Sci.USA.

[6] Riccardi D (1995). Cloning and functional expression of a rat kidney extracellular calcium/polyvalent cation-sensing receptor. Proc. Natl. Acad. Sci.USA.

[7] Sands JM (1997). Apical extracellular calcium/polyvalent cation-sensing receptor regulates vasopressin-elicited water permeability in rat kidney inner medullary collecting duct. J. Clin. Invest..

[8] Tfelt-Hansen J, Brown E (2005). The calcium-sensing receptor in normal physiology and pathophysiology: a review. Crit. Rev. Clin. Lab. Sci..

[9] Chattopadhyay N (1998). Identification and localization of extracellular Ca(2+)-sensing receptor in rat intestine. Am. J. Physiol. Gastrointest. Liver Physiol..

[10] Cheng SX, Geibel J, Hebert S (2004). Extracellular polyamines regulate fluid secretion in rat colonic crypts via the extracellular calcium-sensing receptor. Gastroenterology.

[11] Cheng SX, Okuda M, Hall A, Geibel JP, Hebert SC (2002). Expression of calcium-sensing receptor in rat colonic epithelium: evidence for modulation of fluid secretion. Am. J. Physiol. Gastrointest. Liver Physiol..

[12] Gama L, Baxendale-Cox LM, Breitwieser GE (1997). Ca^2+^-sensing receptors in intestinal epithelium. Am. J. Physiol. Cell Physiol..

[13] Cheng SX (2012). Calcium-sensing receptor inhibits secretagogue-induced electrolyte secretion by intestine via the enteric nervous system. Am. J. Physiol. Gastrointest. Liver Physiol..

[14] Gwynne RM, Ly K, Parry LJ, Bornstein JC (2017). Calcium sensing receptors mediate local inhibitory reflexes evoked by L-phenylalanine in Guinea pig jejunum. Front. Physiol..

[15] Tang L (2016). The Extracellular calcium-sensing receptor in the intestine: evidence for regulation of colonic absorption, secretion, motility, and immunity. Front. Physiol..

[16] Hebert S, Cheng S, Geibel J (2004). Functions and roles of the extracellular Ca^2+^-sensing receptor in the gastrointestinal tract. Cell Calcium.

[17] Sheinin Y (2000). Immunocytochemical localization of the extracellular calcium-sensing receptor in normal and malignant human large intestinal mucosa. J. Histochem. Cytochem..

[18] Geibel J (2006). Calcium-sensing receptor abrogates secretagogue-induced increases in intestinal net fluid secretion by enhancing cyclic nucleotide destruction. Proc. Natl. Acad. Sci.USA.

[19] Furness JB (2012). The enteric nervous system and neurogastroenterology. Nat. Rev. Gastroenterol. Hepatol..

[20] Cooke HJ (2000). Neurotransmitters in neuronal reflexes regulating intestinal secretion. Ann. N. Y. Acad. Sci..

[21] Field M (2003). Intestinal ion transport and the pathophysiology of diarrhea. J. Clin. Invest..

[22] Lundgren O (2002). Enteric nerves and diarrhoea. Pharmacol. Toxicol..

[23] Burleigh DE, Borman RA (1997). Evidence for a nonneural electrogenic effect of cholera toxin on human isolated ileal mucosa. Dig. Dis.Sci..

[24] Lorrot M, Vasseur M (2007). How do the rotavirus NSP4 and bacterial enterotoxins lead differently to diarrhea?. Virol. J..

[25] Lundgren O (2000). Role of the enteric nervous system in the fluid and electrolyte secretion of rotavirus diarrhea. Science.

[26] Cassuto J, Jodal M, Tuttle R, Lundgren O (1981). On the role of intramural nerves in the pathogenesis of cholera toxin-induced intestinal secretion. Scand. J. Gastroenterol..

[27] Cassuto J, Jodal M, Lundgren O (1982). The effect of nicotinic and muscarinic receptor blockade on cholera toxin induced intestinal secretion in rats and cats. Acta Physiol. Scand..

[28] Massy ZA, Henaut L, Larsson TE, Vervloet MG (2014). Calcium-sensing receptor activation in chronic kidney disease: effects beyond parathyroid hormone control. Semin. Nephrol..

[29] Tang L (2015). Calcium-sensing receptor stimulates Cl^–^and SCFA-dependent but inhibits cAMP-dependent HCO_3_^−^ secretion in colon. Am. J. Physiol. Gastrointest. Liver Physiol..

[30] Sun X, Tang L, Winesett S, Chang W, Cheng SX (2017). Calcimimetic R568 inhibits tetrodotoxin-sensitive colonic electrolyte secretion and reduces c-fos expression in myenteric neurons. Life Sci.

[31] Hubel KA (1985). Intestinal nerves and ion transport: stimuli, reflexes, and responses. Am. J. Physiol. Gastrointest. Liver Physiol..

[32] Gabriel SE, Brigman KN, Koller BH, Boucher RC, Stutts MJ (1994). Cystic fibrosis heterozygote resistance to cholera toxin in the cystic fibrosis mouse model. Science.

[33] De Jesus Ferreira MC, Bailly C (1998). Extracellular Ca^2+^ decreases chloride reabsorption in rat CTAL by inhibiting cAMP pathway. Am. J. Physiol. Renal Physiol.

[34] Chen CJ, Barnett JV, Congo DA, Brown EM (1989). Divalent cations suppress 3′,5′-adenosine monophosphate accumulation by stimulating a pertussis toxin-sensitive guanine nucleotide-binding protein in cultured bovine parathyroid cells. Endocrinology.

[35] Nemeth PR, Palmer JM, Wood JD, Zafirov DH (1986). Effects of forskolin on electrical behaviour of myenteric neurones in guinea-pig small intestine. J. Physiol..

[36] Racke K, Schworer H (1991). Regulation of serotonin release from the intestinal mucosa. Pharmacol. Res..

[37] Modlin IM, Kidd M, Pfragner R, Eick GN, Champaneria MC (2006). The functional characterization of normal and neoplastic human enterochromaffin cells. J. Clin. Endocrinol. Metab..

[38] Chang, E. B. & Rao, M. C. in *Physiology of the* Gastrointestinal Tract (ed Johnson, L. R.) 2027–2081 (Raven, 1994).

[39] Conigrave AD, Ward DT (2013). Calcium-sensing receptor (CaSR): pharmacological properties and signaling pathways. Best Pract. Res. Clin. Endocrinol. Metab..

[40] Breitwieser GE (2013). The calcium sensing receptor life cycle: trafficking, cell surface expression, and degradation. Best Pract. Res. Clin. Endocrinol. Metab..

[41] Prince RL, Devine A, Dhaliwal SS, Dick IM (2006). Effects of calcium supplementation on clinical fracture and bone structure: results of a 5-year, double-blind, placebo-controlled trial in elderly women. Arch. Intern. Med..

[42] Ragno A (2012). Chronic constipation in hypercalcemic patients with primary hyperparathyroidism. Eur. Rev. Med. Pharmacol. Sci..

[43] Sagmanligil V, Levin RJ (1993). Electrogenic ion secretion in proximal, mid and distal colon from fed and starved mice. Comp. Biochem. Physiol. C Toxicol. Pharmacol..

[44] Nzegwu HC, Levin RJ (1994). Fluid hypersecretion induced by enterotoxin STa in nutritionally deprived rats: jejunal and ileal dynamics *in vivo*. Exp. Physiol..

[45] Young A, Levin RJ (1992). Intestinal hypersecretion of the refed starved rat: a model for alimentary diarrhoea. Gut.

[46] Young A, Levin RJ (1990). Diarrhoea of famine and malnutrition: investigations using a rat model. 1. Jejunal hypersecretion induced by starvation. Gut.

[47] Young A, Levin RJ (1990). Diarrhoea of famine and malnutrition–investigations using a rat model. 2–Ileal hypersecretion induced by starvation. Gut.

[48] Helweg-Larsen P (1952). Famine disease in German concentration camps; complications and sequels, with special reference to tuberculosis, mental disorders and social consequences. Acta Psychiatr. Neurol. Scand. Suppl..

[49] Levin RJ (1992). The diarrhoea of famine and severe malnutrition–is glucagon the major culprit?. Gut.

[50] Clarke L (2009). A guide to Ussing chamber studies of mouse intestine. Am. J. Physiol. Gastrointest. Liver Physiol..

[51] Cox HM, Cuthbert AW, Hkanson R, Wahlestedt C (1988). The effect of neuropeptide Y and peptide YY on electrogenic ion transport in rat intestinal epithelia. J. Physiol..

[52] Crone SA, Negro A, Trumpp A, Giovannini M, Lee KF (2003). Colonic epithelial expression of ErbB2 is required for postnatal maintenance of the enteric nervous system. Neuron.

[53] Farthing MJ (2000). Enterotoxins and the enteric nervous system - a fatal attraction. Int. J. Med. Microbiol..

[54] Bellono NW (2017). Enterochromaffin cells are gut chemosensors that couple to sensory neural pathways. Cell.

[55] Lauffer JM (1999). Pituitary adenylate cyclase-activating polypeptide modulates gastric enterochromaffin-like cell proliferation in rats. Gastroenterology.

[56] Jones BL, Smith SM (2016). Calcium-sensing receptor: a key target for extracellular calcium signaling in neurons. Front. Physiol..

[57] Frankenhaeuser B, Hodgkin AL (1957). The action of calcium on the electrical properties of squid axons. J. Physiol..

[58] Scheuer T (2011). Regulation of sodium channel activity by phosphorylation. Semin. Cell Dev. Biol..

[59] Miura Y, Naka M, Matsuki N, Nomura H (2012). Differential calcium dependence in basal and forskolin-potentiated spontaneous transmitter release in basolateral amygdala neurons. Neurosci. Lett..

[60] Yoshihara M, Suzuki K, Kidokoro Y (2000). Two independent pathways mediated by cAMP and protein kinase A enhance spontaneous transmitter release at Drosophila neuromuscular junctions. J. Neurosci..

[61] Sinning A, Hubner CA (2013). Minireview: pH and synaptic transmission. FEBS Lett..

[62] Black RE, Brown KH, Becker S (1984). Malnutrition is a determining factor in diarrheal duration, but not incidence, among young children in a longitudinal study in rural Bangladesh. Am. J. Clin. Nutr..

[63] Baqui AH (1993). Cell-mediated immune deficiency and malnutrition are independent risk factors for persistent diarrhea in Bangladeshi children. Am. J. Clin. Nutr..

[64] Fraebel J (2018). Extracellular calcium dictates onset, severity, and recovery of diarrhea in a child with immune-mediated enteropathy. Front. Pediatr..

[65] Bovee-Oudenhoven IMJ, Lettink-Wissink MLG, Van Doesburg W, Witteman BJM, Van Der Meer R (2003). Diarrhea caused by enterotoxigenic *Escherichia coli* infection of humans is inhibited by dietary calcium. Gastroenterology.

[66] Cheng SX, Bai HX, Gonzalez-Peralta R, Mistry PK, Gorelick FS (2013). Calcium ameliorates diarrhea in immunocompromised children. J. Pediatr. Gastroenterol. Nutr..

[67] Speelman P, Butler T, Kabir I, Ali A, Banwell J (1986). Colonic dysfunction during cholera infection. Gastroenterology.

[68] Wickelgren, I. How rotavirus causes diarrhea. *Scienc*e **28**7, 409, 411–409, 411 (2000). 10.1126/science.287.5452.409b 10671159

[69] Wood JD (2006). Histamine, mast cells, and the enteric nervous system in the irritable bowel syndrome, enteritis, and food allergies. Gut.

[70] Margolis KG (2011). Enteric neuronal density contributes to the severity of intestinal inflammation. Gastroenterology.

[71] Rey O, Bikle CW, Rozengurt D, Young N, Rozengurt SH (2012). E. Negative cross-talk between calcium-sensing receptor and β-catenin signaling systems in colonic epithelium. J. Biol. Chem..

[72] Richardson SH, Kuhn RE (1986). Studies on the genetic and cellular control of sensitivity to enterotoxins in the sealed adult mouse model. Infect. Immun..

[73] Gabriel SE (1999). A novel plant-derived inhibitor of cAMP-mediated fluid and chloride secretion. Am. J. Physiol. Gastrointest. Liver Physiol..

[74] Thiagarajah JR, Broadbent T, Hsieh E, Verkman AS (2004). Prevention of toxin-induced intestinal ion and fluid secretion by a small-molecule CFTR inhibitor. Gastroenterology.

